# Evaluation of long-term sequelae by cardiopulmonary exercise testing 12 months after hospitalization for severe COVID-19

**DOI:** 10.1186/s12890-023-02313-x

**Published:** 2023-01-12

**Authors:** Sofia Noureddine, Pauline Roux-Claudé, Lucie Laurent, Ophélie Ritter, Pauline Dolla, Sinan Karaer, Frédéric Claudé, Guillaume Eberst, Virginie Westeel, Cindy Barnig

**Affiliations:** 1 Department of Chest Disease, University Hospital Besançon, 25000 Besançon, France; 2 Methodology and Quality of Life in Oncology Unit, University Hospital, Besançon, France and UMR 1098, University of Franche-Comté, Besançon, France; 3 UMR1098, University of Franche-Comté, INSERM, EFS BFC, F-25000 Besançon, France

**Keywords:** COVID-19, SARS-CoV-2, Acute respiratory distress syndrome, Cardiopulmonary exercise testing, Peak oxygen consumption, Pulmonary vascular disease

## Abstract

**Background:**

Cardiopulmonary exercise testing (CPET) is an important clinical tool that provides a global assessment of the respiratory, circulatory and metabolic responses to exercise which are not adequately reflected through the measurement of individual organ system function at rest. In the context of critical COVID-19, CPET is an ideal approach for assessing long term sequelae.

**Methods:**

In this prospective single-center study, we performed CPET 12 months after symptom onset in 60 patients that had required intensive care unit treatment for a severe COVID-19 infection. Lung function at rest and chest computed tomography (CT) scan were also performed.

**Results:**

Twelve months after severe COVID-19 pneumonia, dyspnea was the most frequently reported symptom although only a minority of patients had impaired respiratory function at rest. Mild ground-glass opacities, reticulations and bronchiectasis were the most common CT scan abnormalities. The majority of the patients (80%) had a peak O_2_ uptake (V′O_2_) considered within normal limits (median peak predicted O_2_ uptake (V′O_2_) of 98% [87.2–106.3]). Length of ICU stay remained an independent predictor of V′O_2_. More than half of the patients with a normal peak predicted V′O_2_ showed ventilatory inefficiency during exercise with an abnormal increase of physiological dead space ventilation (VD/Vt) (median VD/VT of 0.27 [0.21–0.32] at anaerobic threshold (AT) and 0.29 [0.25–0.34] at peak) and a widened median peak alveolar-arterial gradient for O_2_ (35.2 mmHg [31.2–44.8]. Peak PetCO_2_ was significantly lower in subjects with an abnormal increase of VD/Vt (*p* = 0.001). Impairments were more pronounced in patients with dyspnea. Peak VD/Vt values were positively correlated with peak D-Dimer plasma concentrations from blood samples collected during ICU stay (r^2^ = 0.12; *p* = 0.02) and to predicted diffusion capacity of the lung for carbon monoxide (D_LCO_) (r^2^ =  − 0.15; *p* = 0.01).

**Conclusions:**

Twelve months after severe COVID-19 pneumonia, most of the patients had a peak V′O_2_ considered within normal limits but showed ventilatory inefficiency during exercise with increased dead space ventilation that was more pronounced in patients with persistent dyspnea.

*Trial registration*: NCT04519320 (19/08/2020).

**Supplementary Information:**

The online version contains supplementary material available at 10.1186/s12890-023-02313-x.

## Background

In December 2019, Wuhan city identified a new type of coronavirus, named severe acute respiratory syndrome coronavirus 2 (SARS-CoV-2), that rapidly spread all over the world and caused an immense global health crisis. Most patients presented mild to moderate respiratory disease, experiencing cough, fever, headache, myalgia, diarrhea and anosmia. However, around 3–20% of people with SARS-CoV-2 required hospitalization and a considerable subset needed intensive care because of respiratory failure with severe hypoxemia and bilateral radiographic opacities [1].

Studies found that most SARS-CoV-2 survivors, even those who were critically ill during hospital stay, have normal pulmonary function tests within 12 months after symptom onset [2]. Nevertheless, more than half of the patients who recover from Coronavirus disease 2019 (COVID-19) complain of long-term persistent dyspnea [3].

Cardiopulmonary exercise testing (CPET) is a non-invasive clinical tool that provides a global assessment of the respiratory, circulatory and metabolic responses to maximal exercise, which are not adequately reflected through the measurement of individual organ system function at rest [4]. Cycle ergometry is the most common mode of exercise used for CPET. Concomitant to performance assessment, gas exchange, heart and metabolic parameters are analyzed, enabling identification of exercise-limiting factors and pathophysiologic mechanisms involved. In the context of COVID-19, CPET is an ideal approach for unmasking functional anomalies and long-term sequelae. To date, only a few studies have investigated the exercise capacity in patients who have recovered from COVID-19. Assessment in the short-term post-COVID period revealed mostly a mild decrease in peak O_2_ uptake (V′O_2_) and a low anaerobic threshold, without cardiac impairment or ventilatory limitation, suggestive of physical deconditioning [5, 6]. However, there is currently limited data on long term functional capacities in patients after COVID-19, especially after critical infection.

The aim of our study was to evaluate cardiopulmonary exercise capacities in a prospective cohort of patients that required critical care management during the first wave of COVID-19, 12 months after symptom onset.

## Methods

### Study design and subjects

This was a prospective single-center observational cohort study. All patients who were admitted between April to June 2020 to any of the intensive care units (ICU) of the University Hospital of Besançon (France) for a COVID-19 infection were contacted upon hospital discharge and invited to participate in the trial. The study consisted of a follow-up at 3, 6 and 12 months after symptom onset (NCT04519320, 19/08/2020). Cardiopulmonary exercise capacities were only assessed at the 12 months follow-up. Patients were eligible if they were > 18 and < 80 years old and had initially confirmed SARS-CoV-2 infection by quantitative RT-PCR on nasal swabs or bronchoalveolar lavage. Patients were excluded if they were known to have prior chronic respiratory insufficiency, if they had a significant psychiatric pathology, or if they had a life expectancy estimated at less than one year. The protocol was approved by the ethics committee (Comité de Protection des Personnes (CPP) Grand‐Est N° ID RCB: 2020-A01067-32, 21/04/2020) and written informed consent was obtained from all patients at the time of enrollment.

### Pulmonary function tests

Pulmonary function tests were realized in all the patients and included spirometry, measurement of lung volumes by plethysmography and single-breath determination of diffusion capacity of the lung for carbon monoxide (DLCO) (Platinum Elite, MGC Diagnostic Coroporation). Predicted normal values were derived from the reference values in accordance with current recommendations [7, 8]. The modified Medical Research Council (mMRC) dyspnea scale (0 to 4) was used to rate chronic dyspnea [9]. Participants were categorized as having dyspnea (mMRC ≥ 1) or not (mMRC = 0).

### CT image acquisition and analysis

Chest CT scans were acquired in the supine position at full inspiration without contrast medium (Revolution CT; GE Healthcare, Milwaukee, WI, USA). CT images were assessed by two readers blinded to clinical data that evaluated the presence and extent of ground-glass opacities (GGOs), reticulations, bronchiectasis, emphysema and honeycombing as defined by the glossary of terms of the Fleischner Society [10].

### Cardiopulmonary exercise test

Symptom-limited incremental CPET was performed according to the ERS guidelines on an electronically braked cycle ergometer (Ergometrics 900; Ergoline; Bitz, Germany) [11].

After a steady-state resting period, a 3 min warm-up was conducted at about 20% of individually estimated maximal workload. A progressive increase in workload was then applied every minute (10 to 20 W/min) depending on the patient’s physical condition, medical history and according to a total exercise time between 8 and 12 min. Tests were terminated at the point of symptom limitation (peak exercise) or in the presence of electrocardiographic changes. Subjects rated the magnitude of their perceived breathing and leg discomfort by pointing to a number on the 10-point Borg scale [12]. Oxygen saturation with pulse oximetry, heart rate (HR) and 12-lead electrocardiogram (ECG) and non-invasive blood pressure measurements were monitored throughout exercise.

Breath-by-breath gas exchange values were measured using a Masterscreen CPX metabolic cart (MGC-CPX System; MGC Diagnostics Corporation) and were expressed as 30 s averages, according to recommended guidelines. Minute ventilation (V′E), oxygen uptake (V′O_2_), carbon dioxide production (V′CO_2_), end-tidal partial pressure of carbon dioxide (PetCO_2_), tidal volume (Vt) and breathing rate were recorded. Oxygen pulse (V′O_2_/heart rate), ventilatory equivalent for oxygen uptake (V′E/V′O_2_) and ventilatory equivalent for carbon dioxide production (V′E/V′CO_2_) were calculated. The respiratory exchange ratio (RER) was defined as V′CO_2_/V′O_2_. The anaerobic threshold (AT) was determined by both ventilatory equivalents and V-slope methods. V′E/V′CO_2_ slope was calculated from rest to peak exercise.

During CPET, arterialized earlobe blood samples were drawn by a nurse at rest, at anaerobic threshold (AT) and at peak exercise. Partial pressure of oxygen (PaO_2_) and carbon dioxide (PaCO_2_) were measured by a blood gas analyzer (RAPIDPoint® 500, Siemens). Lactatemia was also determined at rest, at AT, and at maximal exercise. Breathing reserve (BR) % was calculated as BR = (predicted maximum voluntary minute ventilation [MMV] − peak VE)/MMV × 100, with predicted MMV = FEV1 × 40. Peak heart rate (HR) was expressed as a percentage of maximum predicted HR, calculated as HR max = 210 − (0.65 × age). Physiological dead space (VD/Vt) was calculated according to Bohr’s equation corrected for the additional instrument dead space: VD/Vt = (PaCO_2_ – PetCO_2_ mean)/PaCO_2_ − (VD [machine]/Vt).

Tests were considered maximal if a plateau of the V′O_2_ > 60 s was obtained (variation of V′O_2_ < 150 mL between 2 increments), RER > 1.1, a perceived exertion > 7 on the Borg scale, peak HR > 100% of predicted, breathing reserve < 15% and/or important metabolic acidosis**.**

Normal predicted values for V′O_2_ were calculated according to the reference equation of Wassermann [13]. A reduced peak exercise capacity was defined by a peak V′O_2_ < 85% of predicted [4].

### Statistical analyses

Statistical analysis was performed using GraphPad Prism version 9.0 (GraphPad Software Inc., San Diego, CA, USA). Normal distribution of quantitative variables was tested by the Kolmogorov–Smirnov test. Descriptive statistics are presented as mean ± standard deviation (SD), median (25th to 75th percentile), or number (%), as appropriate. Student’s T or Mann–Whitney U-test tests were computed to assess statistical differences between groups for normal or non-normal quantitative variables, respectively. Categorical variables were analyzed by Fisher’s exact test when appropriate. Correlations were examined by Spearman rank test or Pearson test. Multiple linear regression was applied for peak V′O_2_ (ml/kg/min) as dependent variable, using a stepwise approach of potential determinants that showed significant associations in previous univariate analysis. Age, sex and body mass index (BMI) were included in the final multivariable model. The reported p values were two-sided, with a significance level set at *p* < 0.05.

## Results

### Baseline characteristics of the study population

From 149 patients that were initially admitted to intensive care with a diagnosis of SARS-CoV-2, a total of 85 patients were included in the cohort study (Additional file 1: Figure 1). Seventy-three patients (86%) completed the 12 months visit that included a clinical evaluation, lung function tests, chest computed tomography (CT) and CPET carried out on the same day. A total of 64 patients performed CPET (2 patients declined to perform CPET, 2 had no negative RT-PCR control for SARS-CoV-2 and 6 had contraindications for performing CPET (pericardial effusion, acid–base disorders, orthopedic pathology and recent head trauma)). Four patients were excluded from the final analysis, 3 because of submaximal efforts and 1 patient that had presented severe arrhythmia during the test leading to an early exercise termination.

The demographics, comorbidities and ICU treatments of included patients are summarized in Table 1. The mean age was 64.6 years (± 9.6), 78% were male. All patients were initially admitted in an intensive care unit (ICU) and were treated according to local standards at that time. The majority of the patients fulfilled criteria for initial ARDS according to the Berlin definition [14], median PaO_2_/FiO_2_ (P/F) ratio at ICU entrance was 157.5 (106.6–213.1) and 12 patients (21%) had P/F ratio < 100, 90% of patients were intubated, 6 (10%) had High Flow Nasal Oxygen (HFNO) before endotracheal intubation, none had noninvasive ventilation (NIV). The median duration of intubation was 16 days (10.7–26.2), 20 patients (33.3%) had HFNO after extubation and 7 (11.7%) NIV, 45% received steroids and more than 90% received anticoagulant therapy within the first 24 h of ICU admission. Computed tomography pulmonary angiography was positive for pulmonary embolism in 27% of the patients during their stay. Peak values during ICU stay for main blood laboratory findings are shown in Table 1. All patients included in the study had early rehabilitation during their hospital stay. More than half of the patients (65%) were further referred to a pulmonary rehabilitation center and most of the other patients had regular home physiotherapy sessions after their hospital discharge.

**Table 1:** Baseline characteristics of the study population

	*n* = 60
Age (years)	64.6 ± 9.6
Male	47 (78%)
BMI (kg/m^2^)	30.7 ± 5
*Smoking status*	
Active smoker	1 (1.7%)
Former smoker	33 (55%)
Never smoker	26 (43.3%)
*Comorbidities before SARS-CoV-2 infection*	
Obesity (BMI > 30)	33 (55%)
Cardiovascular	
Ischemic heart disease	5 (8.3%)
Hypertension	28 (46.7%)
Dyslipidemia	21 (35%)
Diabetes	15 (25%)
Respiratory diseases	
COPD	5 (8.3%)
Asthma	6 (10%)
Sleep apnea	12 (20%)
Thromboembolic disease	
Deep vein thrombosis	3 (5%)
Pulmonary embolism	0 (0.0%)
*Initial hospital management*	
Intensive care unit	
Length of ICU stay (days)	21.2 ± 12.4
SAPS	35.6 ± 9.1**
ARDS^£^	54 (90%)
PaO_2_/FiO_2_	157.5 (106.6–216.1)***
High-flow nasal oxygen before endotracheal intubation	6 (10%)
Endotracheal intubation with mechanical ventilation	54 (90%)
Duration of endotracheal intubation (days)	16 (10.7–26.2)
Neuromuscular blocking agents	53 (89.8%)*
High-dose steroids	27 (45%)
Anticoagulant therapy	55 (91.7%)
Prone position	47 (79.7%)*
Pulmonary embolism	16 (26.7%)
DDimers (ng/ml)	2760 (1601–4507)****
Fibrinogen (g/L)	5.06 ± 1.16
Creatinine (mg/dl)	87.0 (71–117)
CRP (mg/ml)	199.8 ± 94.6
Total WBC count (10^9^/L)	5.4 ± 1.3
Pulmonology unit stay (days)	48.9 ± 36.5
Total length of stay (days)	68.1 ± 45.5
Rehabilitation center	39 (65%)
Home care physiotherapy	9 (15%)

Values are expressed as number of subjects (%), means ± SD or medians [first quartile; third quartile]

*BMI* Body mass index, *COPD* Chronic obstructive pulmonary disease, *ICU* Intensive care unit, *SAPS* Simplified acute physiology score, *ARDS* Acute respiratory distress syndrome, in accordance to the Berlin definition criteria, *CRP* C-reactive protein, *WBC* White blood cell

*Data was unavailable for n = 1 patient

**Data was unavailable for n = 2 patients

***Data was unavailable for n = 4 patients

****Data was unavailable for n = 6 patients

### Characteristics of the study population at 12 months follow-up

The clinical, pulmonary function tests and imaging characteristics of the patients at 12 months follow-up are summarized in Table 2. Dyspnea was reported by half of the patients (50%) (mMRC scale ≥ 1). Only a minority of patients had functional pulmonary impairment at rest. Two patients showed a mild restrictive ventilatory pattern, and 4 patients had airflow obstruction, three of them had a previous diagnosis of COPD. Mildly impaired DLCO, defined as Z-score DLCO < -1.64, was present in 6 patients (10%), 4 were already diagnosed with COPD before SARS-CoV2 infection. When compared to the data from the 3- and 6-months follow-up, lung function overall improved over 12 months (Additional file 2: Table S1). High resolution computed tomography (HRCT) of the chest showed pulmonary abnormalities in 50 patients (84%). Mild ground-glass opacities, reticulations and bronchiectasis were the most common CT scan abnormalities. Cardiac evaluation at rest was only proposed to patients that had presented pulmonary embolism during their hospital stay. In these patients, transthoracic echocardiography was within normal limits with no signs of pulmonary hypertension.

**Table 2:** Characteristics of the study population at 12 months follow-up

	*n* = 60
*Respiratory symptoms*	
Dyspnea	
mMRC 0	30 (50%)
mMRC ≥ 1	30 (50%)
Cough	9 (15%)
*Pulmonary function tests*	
VC (L)	3.7 (3.1–4.3)
VC (% predicted)	109 (95.8–120.5)
FEV_1_ (L)	3.03 (2.5–3.5)
FEV_1_ (% predicted)	106.3 (94.5–123.5)
FEV_1_/VC (%)	81 (72.2–85)
TLC (L)	5.99 (5.1–6.6)
TLC (% predicted)	93 (85.2–103.5)
D_LCO_cor (ml/min/mmHg)	23.8 (19.7–27.1)
D_LCO_cor (% predicted)	99.1 (90.5–112.9)
KCO (ml/min/mmHg/L)	4.4 (9.9–4.8)
KCO (% predicted)	103 (94.3–114.9)
pO_2_ (mmHg)	82.2 ± 9.2
pCO_2_ (mmHg)	36.8 ± 3.9
Bicarbonate (mmol/L)	23.3 ± 2.1
*Chest computed tomography scan*	
Normal	10 (16%)
Reticulations	
1–25%	34 (57%)
26–50%	8 (13%)
> 50%	0 (0.0%)
Ground-glass opacities	
1–25%	27 (45%)
26–50%	1 (1.7%)
> 50%	1 (1.7%)
Bronchiectasis	
1–25%	33 (55%)
26–50%	2 (3.3%)
> 50%	0 (0.0%)
Emphysema	
1–25%	5 (8.3%)
26–50%	3 (5.0%)
> 50%	3 (5.0%)
Honeycombing	
1–25%	3 (5.0%)
> 25%	0 (0.0%)

Data are shown as the number of subjects (%), means ± SD (standard deviation) or medians [first quartile; third quartile]

mMRC, Modified Medical Research Council; FVC, Forced vital capacity; FEV1, Forced expiratory volume at 1st second; TLC, Total lung capacity; D_LCO_cor, Diffusion capacity of carbon monoxide; KCO, Carbon monoxide transfer coefficient; PcapO_2_, Capillary arterialized pO_2_; PcapCO_2_, Capillary arterialized pCO_2_

### Cardiopulmonary exercise test (CPET) results at 12 months follow-up

Adequate exercise test efforts were obtained in all the patients analyzed. Table 3 summarizes the main exercise parameters of the study cohort at the anaerobic threshold (AT) and at peak. Most of the patients had an adequate V′O_2_ at AT (median predicted 64.8% [57.2–70.9]). The median peak predicted V′O_2_ was 98% [87.2–106.3]) (mean peak V′O_2_ 21.7 ± 5.2 mL/min/Kg). Reasons for stopping exercise were leg discomfort in 55% of patients, breathing discomfort in 36.6% patients and both in 5% patients.

**Table 3:** Cardiopulmonary exercise test (CPET) results at 12 months follow-up (n = 60)

Variables	Anaerobic threshold	Peak
*Reasons for stopping*		
Leg discomfort	–	33 (55%)
Breathing discomfort	–	22 (36.6%)
Both	–	3 (5%)
Other	–	2 (3.3%)
*Performance*		
Workload (W, % predicted)	65.8 (57.5–73.2)	103 (92.5–121.3)
V′O_2_ (L/min)	1.3 ± 0.3	1.9 ± 0.5
V′O_2_ (L/min, % predicted)	65 (57.1–70.9)	98.2 (87.2–106.2)
V′O_2_ (ml/min/kg)	14.2 ± 3.1	21.8 ± 5.2
V′O_2_, (ml/min/kg, % predicted)	64.8 (57.2–70.9)	98.0 (87.2–106.3)
V′O_2_/watts (ml/min/watts)	15.3 (14–16.5)	14.3 (13.5–15.7)
MET	4.1 ± 0.9	6.2 ± 1.5
RER	0.9 ± 0.1	1.1 ± 0.1
*Ventilation*		
VT (L)	7.7 ± 0.4	2.2 ± 0.5
VT (% FVC)	46.2 ± 10.9	59.8 ± 8.2
VE (L/min)	39.7 ± 9.7	81.9 ± 22
RR (breaths/min)	24 ± 5	37 ± 6
Breathing reserve (%)	66.7 ± 8.7	32.6 ± 13.7
PetCO_2_ (mmHg)	39.6 ± 5.7*	34.3 ± 5.2*
*Circulation*		
HR (beats/min, % predicted)	78.2 ± 11.2	96.6 ± 12.7
Heart rate reserve (%)	24.3 (12.8–29.9)	2.3 (0–15)
V′O_2_ pulse (ml/beat/min)	10.5 ± 2.6	12.9 ± 3.1
V′O_2_ pulse (% predicted)	84.6 ± 16.1	103.7 ± 19.9
ΔHR/ΔV′O_2_	–	41.9 (33.6–48.7)
ΔV′O_2_/ΔWR	–	14.2 (13.5–15.7)
*Gas exchange*		
V′E/V′O_2_	31.7 ± 4.9	42.7 ± 6.6
V′E/V′CO_2_	33.5 (30–37)	37.5 (34–42)
V′E/V′CO_2_ slope	–	37.2 ± 6.7*
OUES (L/min)	–	1.8 (1.6–2.3)*
pH	7.4 ± 0.03*	7.3 ± 0.05*
pCapO_2_ (mmHg)	82.3 (73.4–85.4)*	85.4 (72.4–87)*
pCapCO_2_ (mmHg)	38.2 ± 4.1*	34.8 ± 4.1*
P(A-a)O_2_ (mmHg)	26.4 (22.4–34.9)*	35.2 (31.2–44.8)*
V_D_/V_T_	0.27 (0.21–0.34)**	0.29 (0.25–0.35)**
*Metabolic*		
Lactatemia (mmol/L)	3.4 ± 1.1*	7.6 ± 2.2**

Data are shown as the number of subjects (%), means ± SD or medians [first quartile; third quartile]

V′O_2_, Oxygen uptake; MET, Metabolic equivalent; RER, Respiratory exchange ratio; VT, Tidal volume; FVC, Forced vital capacity; VE, Minute ventilation; RR, Respiratory rate; PetCO_2_, End-tidal pressure of CO_2_; HR, Heart rate; WR, Work rate; V′E/V′O_2_ and V′E/V′CO_2_, Ventilatory equivalents for oxygen and carbon dioxide; OUES, Oxygen uptake efficiency slope; PcapO_2_, Capillary arterialized pO_2_; PcapCO_2_, Capillary arterialized pCO_2_; P(A-a)O_2_, Alveolar-arterial gradient for O_2_; VD, Dead space

*Missing values for n = 3 patients

**Missing values for n = 1 patient

Circulatory parameters revealed a mean peak predicted oxygen pulse at 103.7% (± 19.9). Only 8 patients had a mildly decreased O_2_ pulse, 3 of them were under betablockers. The mean peak predicted heart rate was 96.6% (± 12.7), and the median HR/V′O_2_ slope was in the limit of normal at 41.9 (33.6–48.7).

Mean respiratory equivalents at peak were slightly elevated compared to expected values (V′E/V′O_2_ ratio at 42.7 [± 6.6] and V′E/V′CO_2_ ratio at 37.5 [34–42]) and the mean V′E/V′CO_2_ slope from rest to peak was also slightly skewed to increased values compared to expected values (37.2 ± 6.7) [15]. There was also a trend to a widened median alveolar-arterial O_2_ pressure difference at peak (35.2 mmHg [31.2–44.8]).

### Predictors of peak oxygen uptake

**Fig. 1:**
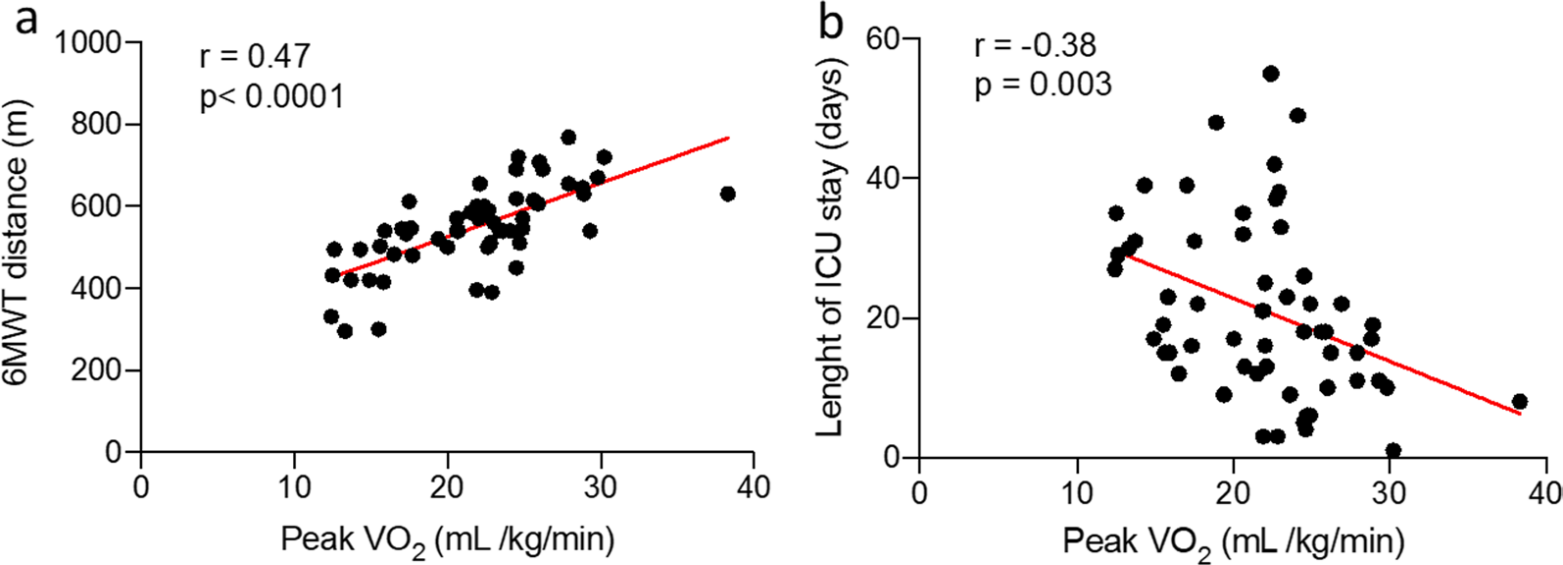
Scatterplot depicting the relationship of peak oxygen uptake (V′O_2_) with **a** 6-min walk test (MWT) distance, **b** length of ICU stay (days)

We next examined the relationship between peak V′O_2_ (ml/min/kg) and variables of interests. Univariate analysis revealed that peak V′O_2_ was strongly correlated to the 6-min walk test (MWT) distance recorded at 12 months (Fig. 1a). As expected, absolute values of lung function test parameters at 12 months (FEV_1_, VC and D_LCO_) were significantly associated to peak V′O_2_ (Additional file 3: Table S2).

Among variables recorded during the management of the acute COVID-19 infection, length of ICU stay showed the most significant correlation with peak V′O_2_ (Fig. 1b). Simplified acute physiology score (SAPS II) and length of curarization had a weaker correlation with peak V′O_2_ (Additional file 3: Table S2).

In a multiple linear regression analysis, the length of ICU stay remained an independent predictor of V′O_2_ and combined to age, sex, and BMI explained 57% of the variance of V′O_2_ peak at 12 months (Table 4).

**Table 4:** Multiple linear regression identifying factors associated with V′O_2_ peak (ml/kg/min)

	*p* value	β coefficient	95% CI for β	Standard error of β
Intercept	< 0.0001	53.60	44.07 to 63.13	4.75
Age (years)	< 0.001	− 0.19	− 0.23 to − 0.08	0.05
Sex	< 0.001	− 4.36	− 6.85 to − 1.90	1.23
BMI	< 0.001	− 0.42	− 0.65 to − 0.19	1.11
Length of ICU stay (days)	0.01	− 0.10	− 0.18 to − 0.02	0.04

*R*^2^ 0.57

### Comparison of patients with reduced and normal exercise capacity

Twelve patients (20%) had reduced peak exercise capacity (V′O_2_ < 85% of predicted) (Table 5). The median peak predicted V′O_2_ (82% [73.9–83.9]) and workload (85.7% [80.1–96.9]) were only mildly decreased. When compared to patient with preserved peak exercise capacity, patients with reduced capacity had a significantly higher BMI (33.4 ± 6.2 vs 30.1 ± 4.5, *p* = 0.04) and a significantly longer ICU stay (29.7 ± 13.1 vs 19.1 ± 11.3 *p* = 0.006). No significant differences were observed between both groups regarding age, other prior comorbidities, pulmonary embolism during ICU stay and respiratory rehabilitation. No significant differences were observed for CT scan abnormalities. However, patients with a reduced exercise capacity had a significantly lower % predicted FEV1 (97.4 ± 16.9 vs 110.8 ± 19.7; *p* = 0.03), FVC (97.5 ± 17.8 vs 110.8 ± 16.9, *p* = 0.01) and a lower % predicted D_LCO_ (91.3 (65.6–98.1) vs 103 (92.3–114.2); *p* = 0.01). Assessment of each individual with limited peak exercise capacity revealed that the primary limitation was ventilatory limitation in 6 patients (50%). Among patients with ventilatory impairment, 5 of them were former smokers and had prior COPD and/or lung emphysema on chest CT. Physical deconditioning was observed for the 6 (50%) others patients.

**Table 5:** Comparison of patients with reduced and normal exercise capacity

Variables	VO_2_ peak < 85% (n = 12)	VO_2_ peak ≥ 85% (n = 48)	*p* value
*Patient’s characteristics*			
Age (years)	65.4 (59.8–72.3)	68.1 (58.1–71.8)	0.96
BMI (kg/m^2^)	33.4 ± 6.2	30.1 ± 4.5	**0.04**
Smoker or former smoker	8 (66.7%)	26 (54.2%)	0.52
ICU stay (days)	29.7 ± 13.1	19.1 ± 11.3	**0.006**
Ischemic heart disease	1 (8.3%)	4 (8.3%)	> 0.99
COPD	1 (8.3%)	4 (8.3%)	> 0.99
Asthma	2 (16.7%)	4 (8.3%)	0.59
Pulmonary embolism	3 (25%)	13 (27.1%)	0.88
Respiratory rehabilitation	9 (75%)	30 (62.5%)	0.41
*Chest computed tomography scan #*			
Emphysema	4 (33.3%)	7 (14.6%)	0.69
Reticulations	9 (75%)	33 (68.7%)	> 0.99
Traction bronchiectasis	8 (66.7%)	27 (56.2%)	0.74
Honeycombing	0 (0.0%)	3 (6.2%)	> 0.99
Ground-glass opacities	4 (33.3%)	25 (52.1%)	0.24
*Pulmonary function tests*			
FEV1 (% predicted)	97.4 ± 16.9	110.8 ± 19.7	**0.03**
FVC (% predicted)	97.5 ± 17.8	110.8 ± 16.9	**0.01**
TLC (%predicted)	87.7 ± 12.6	95.9 ± 14.3	0.07
DL_CO_cor (%predicted)	91.3 (65.6–98.1)	103 (92.3–114.2)	**0.01**
KCO (%predicted)	96.1 ± 29.9	104.8 ± 15.5	0.16
*CPET at AT*			
PetCO_2_ (mmHg)	39.3 ± 6.9 *	39.6 ± 5.5 **	0.87
Anaerobic threshold (%VO_2_ peak predicted)	59.1 (49.1–65.2)	66.1 (58.1–73.2)	**0.01**
*CPET at peak*			
Performance			
Reasons for stopping exercise			
Leg discomfort	8 (66.7%)	25 (52.1%)	0.51
Dyspnea discomfort	4 (33.3%)	18 (37.5%)	> 0.99
Both	0 (0.0%)	3 (6.2%)	> 0.99
Others	0 (0.0%)	2 (4.2%)	> 0.99
Effort duration (sec)	547.6 ± 128.9	585.7 ± 86.2	0.21
Workload (% predicted)	85.7 (80.1–96.9)	107.2 (98.4–124.5)	** < 0.0001**
V′O_2_ (L/min)	1.6 ± 0.5	2.0 ± 0.5	**0.03**
V′O_2_ (L/min, % predicted)	82.1 (73.7–83.7)	101.9 (94.6–107.7)	** < 0.0001**
V′O_2_ (ml/min/kg)	16.9 ± 4.2	23.0 ± 4.7	**0.0001**
V′O_2_ (ml/min/kg, % predicted)	82 (73.9–83.9)	101.6 (94.8–107.5)	** < 0.0001**
MET	4.8 ± 1.2	6.6 ± 1.3	**0.0001**
*Ventilation*			
VE (L/min)	74.9 ± 21.9	83.6 ± 21.9	0.22
RER	1.1 ± 0.1	1.1 ± 0.1	0.14
Breathing reserve (%)	34.4 ± 15.4	32.1 ± 13.4	0.61
PetCO_2_ (mmHg)	34.0 ± 5.3 *	34.3 ± 5.3 **	0.86
*Circulation*			
HR (beats/min, %predicted)	94.3 ± 11.9	97.2 ± 12.9	0.49
Heart rate reserve (%)	4.1 (0–18.6)	1.6 (0–14.7)	0.68
VO_2_ pulse (%predicted)	83.9 ± 11.3	108.7 ± 18.6	** < 0.0001**
ΔHR/ΔV′O_2_	47.5 (36.6–51.2)	39.9 (32.2–47.5)	0.08
ΔV′O_2_/ΔWR	13.9 (12.9–16.1)	14.3 (13.6–15.3)	0.54
*Gas exchange*			
VE/VO_2_ ratio	45.7 ± 7.9	42.1 ± 6.1	0.08
OUES (L/min)	1.6 (1.5–2.0)	1.9 (1.7–2.3)	0.14
VE/VCO_2_ ratio	39.5 (33.5–47.0)	37.5 (34–41)	0.36
VE/VCO_2_ slope	37.2 ± 7.6*	37.2 ± 6.6*	0.99
VD/Vt	0.35 (0.28–0.40)*	0.29 (0.25–0.34)**	0.06
pH	7.3 ± 0.04**	7.3 ± 0.05	0.36
pCapO_2_ (mmHg)	78.2 (54.2–82.1)*	83.8 (76.8–87.1)**	0.05
pCapCO_2_ (mmHg)	35.9 (33.4–39.4)**	34.7 (30.7–37.3)	0.14
P(A-a) (mmHg)	43.49 (29.7–60.5)*	35.2 (31.2–44.8)**	0.23
*Metabolic*			
Lactatemia (mmol/L)	7.2 ± 2.4*	7.7 ± 2.2	0.54

Data are shown as the number of subjects (%), means ± SD or medians [first quartile; third quartile], Student’s t- or Mann–Whitney tests were computed to assess statistical differences for normal or non-normal quantitative. Fisher’s exact test was used for analysis of contingency tables

BMI, Body mass index; ICU, Intensive care unit; COPD: Chronic obstructive pulmonary disease; FEV1, Forced expiratory volume at 1st second; FVC, Forced vital capacity; TLC, Total lung capacity; DLCOcor, Lung transfer for carbon monoxide; KCO, Carbon monoxide transfer coefficient; V′O_2_, Oxygen uptake; MET, Metabolic equivalent; RR, Respiratory rate; V′E, Minute ventilation; RER, Respiratory exchange ratio; PetCO_2_, End-tidal pressure of CO_2_; HR, Heart rate; WR: Work rate; V′CO_2_, Carbon dioxide production; OUES, Oxygen uptake efficiency slope; V′E/V′O_2_ and V′E/V′CO_2_, Ventilatory equivalents for oxygen and carbon dioxide; VD, Dead space; Vt, Tidal volume; PcapO_2_, Capillary arterialized pO_2_; PcapCO_2_, Capillary arterialized pCO_2_; P(A-a)O_2_; Alveolar-arterial gradient for O_2_

*Missing values for n = 2 patients

**Missing values for n = 1 patient

Bold represents *p*-values that are statistically significant

In the group of patients having an exercise capacity considered within normal limits, the median peak predicted V′O_2_ was 101.6% [94.8–107.5] (mean of 23.0 ml/kg/min [± 4.7]) (Table 5). The main reasons for termination were leg discomfort in 52.1% of patients and dyspnea in 37.5%. Despite having a normal exercise capacity, it was worth noting that patients had increased mean ventilatory equivalents for CO_2_ with a mean peak V′E/V′CO_2_ at 37.5 (34–41) and a mean V′E/V′CO_2_ slope from rest to peak at 37.2 [± 6.6]. The median median alveolar-arterial O_2_ pressure difference at peak appeared also widened (34.7 mmHg [31.7–43.9]).

### Ventilatory efficiency parameters in patients with normal exercise capacity

As we observed that a majority of patients with normal exercise capacity showed elevated mean ventilatory equivalents for CO_2_ during exercise, we next focused on the evolution of ventilatory efficiency parameters in this group.

**Fig. 2:**
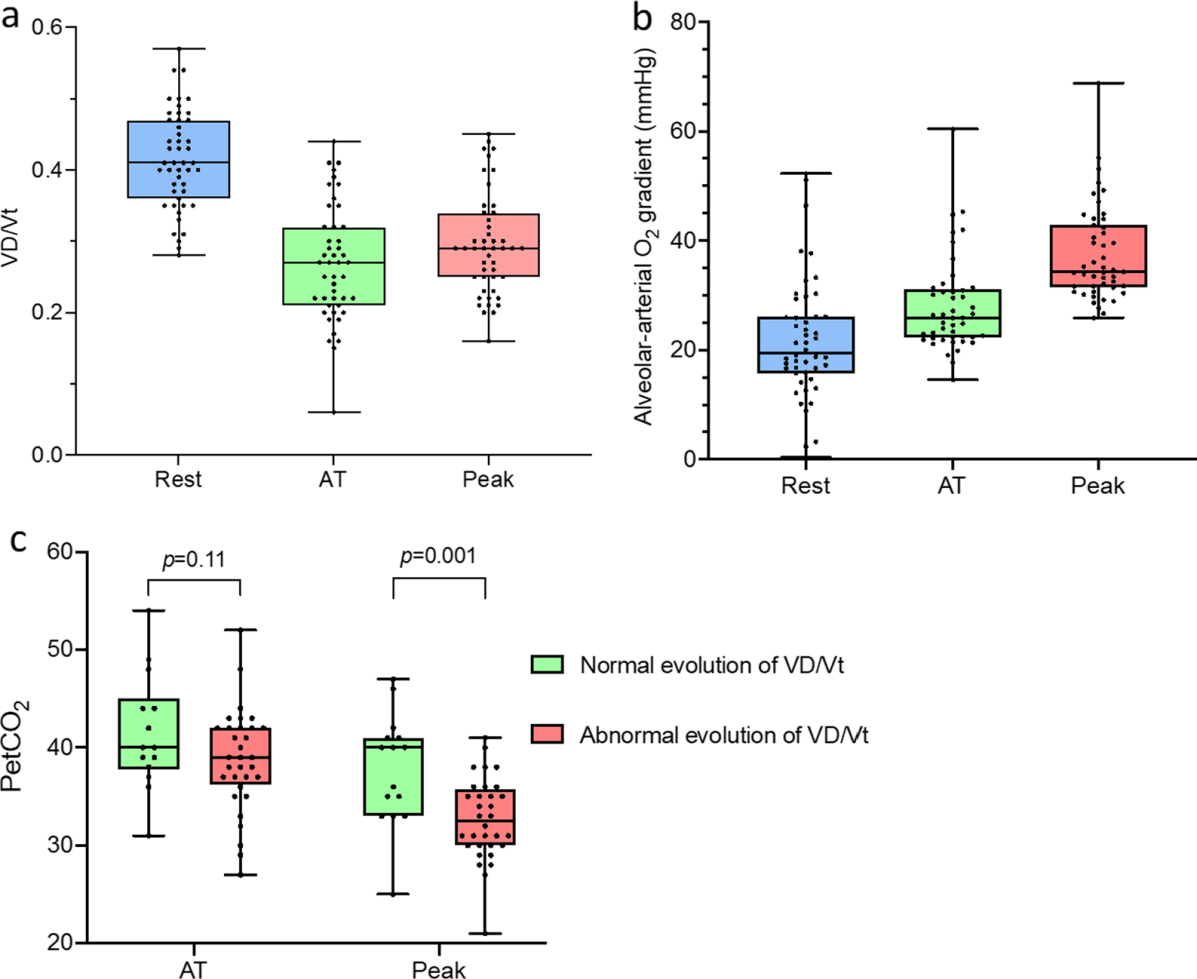
Evolution of ventilatory efficiency parameters from rest to peak in patients with normal exercise capacity (n = 48). **a** VD/Vt, **b** alveolar-arterial gradient, **c** PetCO_2_

It was worth noting that at AT, 18 of those 48 patients (37.5%) had a V′E/V′CO_2_ ratio > 35 and 7 patients (14.6%) had even a V′E/V′CO_2_ ratio > 40. At peak exercise, 56.2% (n = 27) of patients had a V′E/V′CO_2_ slope > 35 and 41.7% (n = 20) had a V′E/V′CO_2_ slope > 40. Anarchical evolution of tidal volume was not observed. In contrast, elevated ventilatory equivalents for CO2 were associated with abnormal dead space ventilation. Indeed, an abnormal increase of the physiological dead space from AT to peak was observed in 68.1% (n = 32) of the patients with a median VD/Vt of 0.27 (0.21–0.32) at AT and 0.29 (0.25–0.34) at peak (Fig. 2a). Moreover, the median peak alveolar-arterial gradient for O_2_ was abnormally elevated (35.2 mmHg [31.2–44.8]) with 48.9% of patients (n = 23) having a P(A-a) ≥ 35 mmHg (Fig. 2b). PetCO2 at peak was significantly lower in subjects with an abnormal increase of VD/Vt (*p* = 0.001) (Fig. 2c).

Univariate analysis revealed that dead space at peak (VD/Vt) was associated with parameters related to pulmonary exchange capacity at rest (pulmonary diffusing capacity) and during exercise (V′E/V′CO_2_ ratio and slope, PetCO_2_, pO_2_ and alveolar-arterial gradient (Table 6). Notably, dead space at peak was positively correlated to D-Dimer plasma concentration from blood samples collected during ICU stay. Reanalysis after excluding patients with pulmonary embolism during ICU stay did not alter this correlation.

**Table 6:** Results of univariate analysis to identify factors associated with peak VD/Vt in patients with normal exercise capacity (n = 48)

	r^2^	*p* value
*Patient’s characteristics*		
Age (years)	0.13	**0.01**
Sexe	0.07	0.07
BMI (kg/m^2^)	0.03	0.2
Length of ICU stay (days)	0.24	0.10
Pulmonary embolism	0.01	0.3
CRP (mg/ml)	0.004	0.6
Fibrinogen (g/L)	0.009	0.5
D-Dimers (ng/ml)	0.12	**0.02**
Creatinine (mg/dl)	0.07	0.07
Total WBC count (10^9^/L)	0.03	0.2
*Pulmonary function tests*		
FEV1 (% predicted)	0.08	0.05
FEV1 (L)	0.02	0.3
VC (% predicted)	0.03	0.2
VC (L)	0.002	0.7
DL_CO_ cor (%predicted)	− 0.15	**0.01**
DL_CO_ cor (ml/min/mmHg)	− 0.06	0.09
*CPET at AT*		
PetCO_2_	− 0.53	**0.0001**
%VO_2_ peak predicted	− 0.11	0.46
*CPET at peak*		
V′O_2_ peak, ml/kg/min %predicted	− 0.17	0.25
V′O_2_ peak, ml/kg/min		
PetCO_2_	− 0.43	**0.003**
V′E/VO_2_ ratio	0.48	**0.0006**
V′E/V′CO_2_ ratio	0.58	** < 0.0001**
V′E/V′CO_2_ slope	0.53	**0.0001**
V′O_2_ pulse (%predicted)	0.1	0.52
pO_2_ (mmHg)	− 0.13	**0.01**
pCO_2_ (mmHg)	0.004	0.8
P(A-a) (mmHg)	0.46	**0.01**

BMI, Body mass index; ICU, Intensive care unit; CRP, C-reactive protein; WBC, White blood cell; FEV1, Forced expiratory volume at 1st second; FVC, Forced vital capacity; TLC, Total lung capacity; D_LCO_cor, Lung transfer for carbon monoxide; KCO, Carbon monoxide transfer coefficient; PetCO_2_, End-tidal pressure of CO_2_; RER, Respiratory exchange ratio; V′O_2_, Oxygen uptake; V′E/V′O_2_ and V′E/V′CO_2_, Ventilatory equivalents for oxygen and carbon dioxide; HR, Heart rate; PcapO_2_, Capillary arterialized pO_2_; PcapCO_2_, Capillary arterialized pCO_2_;P(A-a)O_2_, Alveolar-arterial O_2_ gradient

Bold represents *p*-values that are statistically significant

Interestingly, patients with dyspnea (n = 23) had a significantly higher mean peak dead space (0.32 ± 0.07 vs 0.28 ± 0.06; *p* = 0.04) and a higher widening of their mean peak alveolar-arterial gradient (40.9 ± 9.8 vs 34.2 ± 5.9; *p* = 0.006) (Additional file 3: Table S2). There was no significant difference for the mean peak VE/VCO_2_ ratio and the mean VE/VCO_2_ slope. Performance and circulatory parameters during exercise were also similar between both groups. As expected, breathlessness was the predominant symptom that resulted in test termination in patients with dyspnea. Patients with dyspnea were slightly older. No differences were observed for any other parameters recorded during initial hospitalization including the presence of pulmonary embolism between patients with and without dyspnea. Lung function at rest was also similar at 12 months between both groups (Additional file 4: Table S3).

## Discussion

This prospective study assessed cardiopulmonary exercise performance in 60 patients, 12 months after a critical COVID-19 infection during the first wave that required initial ICU management. The patients of our cohort presented well-established risk factors for severe COVID-19 such as advanced age, a predominance of male sex and a high BMI [16]. As expected, hypertension and dyslipidemia were the most frequent chronic comorbidities.

Despite the severity of the initial clinical presentation, exercise capacity assessed by CPET were within normal limits in most of the patients 12 months after the acute infection. Impairment was predominantly related to persistent deconditioning or prior respiratory comorbidities. These results confirm previous studies assessing exercise capacity by CPET 3 to 6 months after hospital release and reporting that remaining exercise limitation after COVID-19 is primarily related to physical deconditioning rather than to physiological impairment [5, 6, 17, 18]. Thus, recovery of physical capacities after a critical COVID-19 infection appears better than in patients with other ARDS etiologies [19, 20].

In our study, 12 months after the acute infection, the length of ICU stay was still an independent predictor of peak V′O_2_, including the patients that had recovered a peak V′O_2_ considered within normal limits. Even if other studies have already reported associations between length of ICU stay after COVID-19 and peak V′O_2_ [21, 22], we were surprised that this association remained still true several months after hospital release. This was even more unexpected as all our patients had received early physiotherapy management in the acute hospital setting followed by either inpatient rehabilitation or extensive physiotherapy for several weeks at home for most of them.

Another intriguing observation in our study was that many patients having a peak V′O_2_ within normal limits and normal rest lung function, showed ventilatory inefficiency during exercise, with increased V′E/V′CO_2_ ratios at AT and at peak associated with an increased V′E/V′CO_2_ slope. Ventilatory inefficiency after acute COVID-19 has already been reported but mainly attributed to dysfunctional breathing with inappropriate hyperventilation [23]. In contrast to our study, these studies enrolled predominantly patients having had mild COVID-19 [21, 24–26].

In our patients, there was no evidence of exaggerated hyperventilatory response. Indeed, we did not see abnormal respiratory alkalosis, nor anarchical evolution of tidal volume. However, ventilatory inefficiency was associated in our study with increased physiological dead space ventilation. Indeed, we observed that nearly two-thirds of the patients with a normal peak predicted V′O_2_ exhibited an increase of the VD/Vt ratio between AT and peak exercise. Ventilatory inefficiency without hyperventilation syndrome has been suggested by other groups but these studies included patients with a range of disease severity combining data from outpatients, hospitalized patients, and those who had required admission to the ICU [22, 27]. Of interest, Ambrosino et al*.* identified in a study that included mostly severe-to-critical COVID-19 patients without any prior history of cardiovascular or pulmonary disease shortly after hospital release, higher V′E/V′CO_2_ ratios and V′E/V′CO_2_ slopes and a lower VD/Vt decrease among patients with reduced exercise capacity [28].

In healthy individuals, VD/Vt decreases usually during exercise as Vt increases several folds and to a much greater extent than the small increase in VD [29]. Thus, an increase of physiological dead space ventilation during exercise is usually considered as abnormal and may suggest the presence of several cardiac and pulmonary disorders. Increasing VD/Vt during exercise can also be sensitive for pulmonary vascular disease, in particular when associated to low PetCO_2_ [30–32]. As in our study the majority of patients with normal exercise capacity had normal rest pulmonary function, and no cardiovascular abnormalities, the increase of physiological dead space ventilation associated with a low peak PetCO_2_ may therefore point to pulmonary microvascular disease.

It is now apparent that SARS-CoV-2 infection induces endothelial cell dysfunction with systemic inflammatory response resulting in a prothrombotic state manifesting especially with microthrombosis [1]. Notably, in our study, peak VD/Vt values at 12 months were positively correlated to peak D-Dimers plasma concentrations from blood samples collected during ICU stay. D-Dimers are a strong biomarker for hypercoagulability and thrombotic events and can be linked to endothelial dysfunction reported during acute COVID-19 [33, 34]. Therefore, the observed ventilatory inefficiency in our patients may point to infra-clinical pulmonary vasculopathy sequelae due to lung micro-thrombotic events during acute SARS-CoV-2 infection. In a study aiming to quantify endothelial alterations in 23 patients with moderate to critical COVID-19, sublingual video microscopy confirmed microcirculatory alterations that were closely associated with D-Dimer levels [35]. More recently, in a cohort of severe-to-critical COVID-19 patients, Ambrosino et al*.* showed that persistent endothelial dysfunction explored by ultrasound assessment of endothelium-dependent flow-mediated dilation (FMD) was correlated to ventilatory inefficiency parameters during CPET in a subgroup of patients [28]. A recent study in critical COVID-19 patients requiring invasive mechanical ventilation reported the presence of early CT scan signs of microvascular involvement such as vascular enlargement pattern and vascular tree in bud [36]. In this study, extended microvascular signs were significantly more frequent among non-responders to prone positioning [36].

It was interesting to note, that 12 months after the severe COVID-19 pneumonia, more than 80% of the patients showed still pulmonary abnormalities on CT chest. Mild ground-glass opacities, reticulations and bronchiectasis were most common and honeycombing was only seen in 3 patients. Therefore, underlying parenchymal microscopic fibrosis may also contribute to our observations. Indeed, even if the majority of patients had a diffusion capacity at rest considered within normal limits 12 months after the acute infection, peak VD/Vt was significantly correlated to predicted D_LCO_.

Half of our patients, including those who had an exercise capacity within normal limits, still complained of dyspnea. A recent study evaluating the health-related quality of life and persistent symptoms in critically ill COVID-19 patients at twelve months identified a similar proportion with 58.4% of patients with persistent mild dyspnea that was weakly correlated with both DLCO and length of invasive mechanical ventilation [37]. In our study, we found no clear association between dyspnea, length of ICU stay, effort capacity or rest lung function parameters. However, patients with dyspnea had significantly higher mean peak dead space associated to a higher widening of their mean peak alveolar-arterial gradient during exercise. A recent study also reported normal rest pulmonary function tests in patients experiencing still dyspnea 12 months after hospital discharge for COVID-19 pneumonia [38]. These patients showed significant higher levels of ventilation inhomogeneity in both resting and forced breathing [38]. In our study, a potential contribution of impaired dynamic of regional ventilation in the cause of the dyspnea cannot be excluded.

Some potential limitations of our study should be noted. Our study was conducted in a single center. There was also a missing baseline assessment of dyspnea and cardiopulmonary function at rest before SARS-CoV-2 infection in our patients. Even if nearly all the patients with normal exercise capacity and ventilatory inefficiency at exercise had spirometry and predicted DLCO within normal limits, it would have been interesting to compare the results with matched controls. Indeed, more than half of our study cohort were smokers or former smokers. Moreover, we were not able to measure the diffusing capacities of the lung for nitric oxide (NO) which combined to D_LCO_ would have been useful to evaluate more precisely the pulmonary vascular implication. Finally, all patients of our cohort were treated according to local standards at the time of the first wave of COVID-19. Consequently, only 45% of them received corticosteroids but notably all of the patients were precociously anticoagulated during their ICU stay.

## Conclusion

In the current study we report that 12 months after severe COVID-19, the length of the ICU stay remained a significant predictor of peak V′O_2_. Moreover, more than half of the patients that had a peak V′O_2_ considered within normal limits showed ventilatory inefficiency during exercise with increased dead space ventilation that was more pronounced in patients with persistent dyspnea. Further studies are required to clarify our observations.

## Supplementary Information

**Additional file 1. Figure S1.** Flowchart.

**Additional file 2. Table S1.** Pulmonary function tests at 3, 6 and 12 months follow up.

**Additional file 3. Table S2.** Results of univariate analysis to identify factors associated with V′O_2_ peak (ml/kg/min).

**Additional file 4. Table S3.** Comparison of patients with and without persistent dyspnea and normal exercise capacity.

**Additional file 5. Table S4.** Raw data.

## Data Availability

The raw datasets used during the current study is joined in a Additional file 5 (Table S4).

## Abbreviations

ARDS: Acute respiratory distress syndrome
AT: Anaerobic threshold
BMI: Body mass index
COPD: Chronic obstructive pulmonary disease
COVID-19: Coronavirus disease 2019
CPET: Cardiopulmonary exercise testing
CRP: C-reactive protein
CT: Chest computed tomography
D_LCO_: Diffusion capacity of the lung for carbon monoxide
FEV1: Forced expiratory volume at 1st second
FVC: Forced vital capacity
HR: Heart rate
ICU: Intensive care unit
KCO: Carbon monoxide transfer coefficient
MET: Metabolic equivalent
mMRC: Modified Medical Research Council
OUES: Oxygen uptake efficiency slope
P(A-a)O_2_: Alveolar-arterial gradient for O_2_
PcapO_2_: Capillary arterialized pO_2_
PcapCO_2_: Capillary arterialized pCO_2_
PETCO_2_: End-tidal CO_2_ partial pressure
V′O_2_: O_2_ uptake
RER: Respiratory exchange ratio
RR: Respiratory rate
SARS-CoV-2: Acute respiratory syndrome coronavirus 2
TLC: Total lung capacity
VD: Dead space
VE: Minute ventilation
V′E/V′O_2_ and V′E/V′CO_2_: Ventilatory equivalents for oxygen and carbon dioxide
VT: Tidal volume
WBC: White blood cell
WR: Work rate
