# Supplementary figures and images for: Evaluation of long-term sequelae by cardiopulmonary exercise testing 12 months after hospitalization for severe COVID-19
Source: BMC Pulm Med. 2023 Jan 12;23:13. doi: 10.1186/s12890-023-02313-x (PMC9834678; doi:10.1186/s12890-023-02313-x)

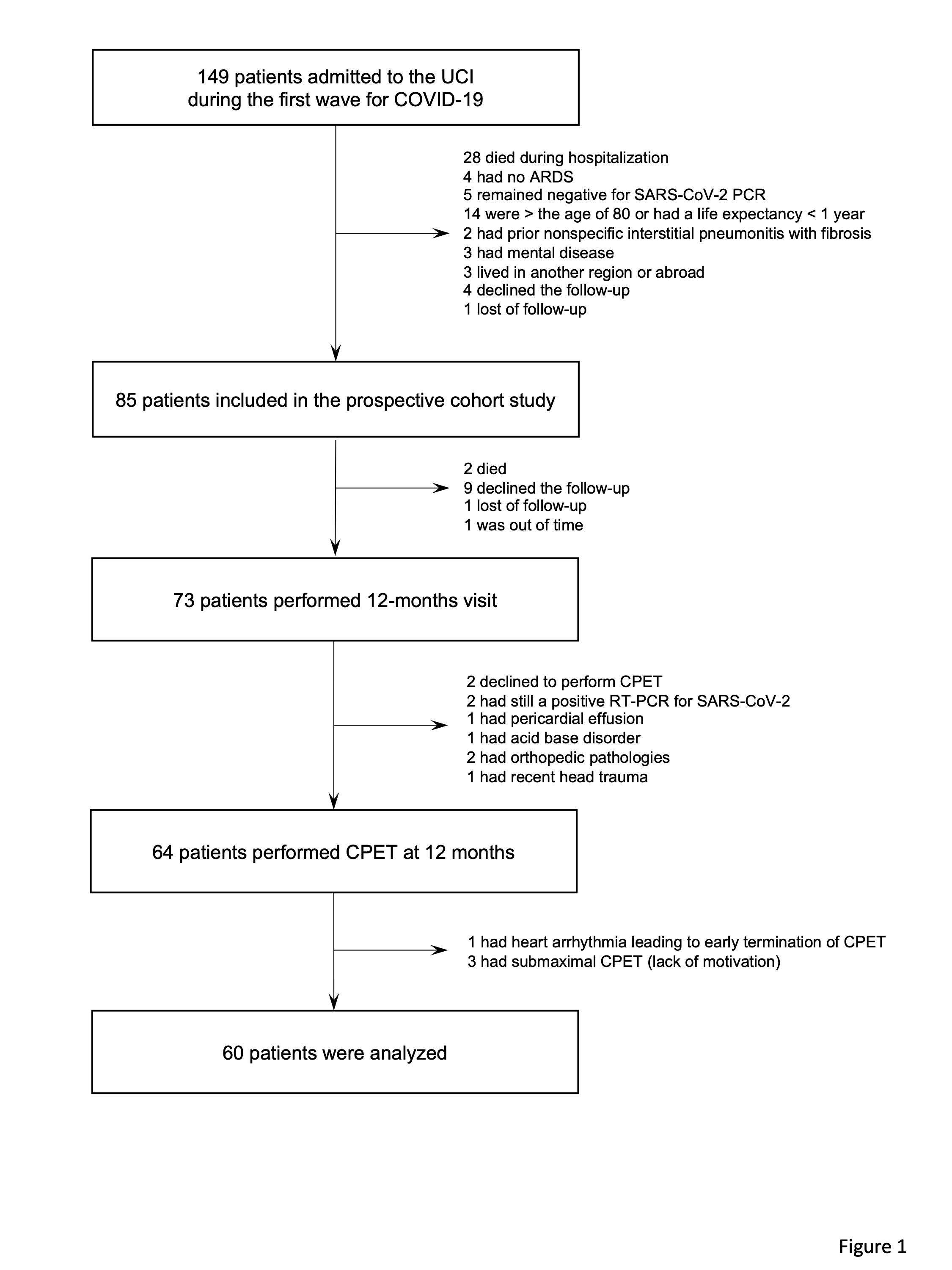

Supplement: Supplementary file 1 — Additional file 1. Figure S1. Flowchart. [file 12890_2023_2313_MOESM1_ESM.jpg]
