# Supplementary material for: Evaluation of long-term sequelae by cardiopulmonary exercise testing 12 months after hospitalization for severe COVID-19
Source: BMC Pulm Med. 2023 Jan 12;23:13. doi: 10.1186/s12890-023-02313-x (PMC9834678; doi:10.1186/s12890-023-02313-x)
Supplement: Supplementary file 2 — Additional file 2. Table S1. Pulmonary function tests at 3, 6 and 12 months follow up. [file 12890_2023_2313_MOESM2_ESM.docx]

**Suppl Table 1.** Pulmonary function tests at 3, 6 and 12 months follow up.

| n = 60 |  |  | M3 | M6 | M12 |
| --- | --- | --- | --- | --- | --- |
| **Respiratory symptoms** | | | | | |
|  | Dyspnea | |  |  |  |
|  |  | mMRC 0 | 22 (36.7%) | 31 (51.7%) | 30 (50%) |
|  |  | mMRC ≥ 1 | 38 (63.3%) | 29 (48.3%) | 30 (50%) |
|  | Cough | | 8 (13.3%) | 3 (5%) | 9 (15%) |
| **Pulmonary function tests** | | |  | | |
|  |  | VC (L) | 3.5 (2.6-4.1) | 3.7 (3.2-4.4) | 3.7 (3.1-4.3) |
|  |  | VC (% predicted) | 94.8 (81.7-104) | 101.6 (90.5-114.5) | 109 (95.8-120.5) |
|  |  | FEV_1_ (L) | 2.8 (2.2-3.2) | 3.0 (2.4-3.3) | 3.03 (2.5-3.5) |
|  |  | FEV_1_ (% predicted) | 96 (85.5-109.1) | 102.7 (93.3-116) | 106.3 (94.5-123.5) |
|  |  | FEV_1_/VC (%) | 82.5 (77-87.5 | 81 (75-85.7) | 81 (72.2-85) |
|  |  | TLC (L) | 5.5 (4.6-6.5) | 6 (5-6.8) | 5.99 (5.1-6.6) |
|  |  | TLC (% predicted) | 89.5 (77-99.5) | 95 (85.5-103) | 93 (85.2-103.5) |
|  |  | D_LCO_cor (ml/min/mmHg) | 18 (15-22.5) | 20.9 (18.3-25.8) | 23.8 (19.7-27.1) |
|  |  | D_LCO_cor (% predicted) | 80 (64.8-94.3) | 90.6 (79.4-105.7) | 99.1 (90.5-112.9) |
|  |  | KCO (ml/min/mmHg/L) | 3.9 (3.2-4.6) | 4.1 (3.6-4.7) | 4.4 (9.9-4.8) |
|  |  | KCO (% predicted) | 88.8 (76.1-108) | 97.9 (85.8-109.5) | 103 (94.3-114.9) |

Data are shown as the number of subjects (%), means ± SD (standard deviation) or medians [first quartile; third quartile]. Abbreviations: mMRC, modified Medical Research Council; FVC, Forced Vital Capacity; FEV1, Forced Expiratory Volume at 1st second; TLC, Total Lung Capacity; D_LCO_cor, Diffusion Capacity of carbon monoxide; KCO, Carbon monoxide transfer coefficient.
